# Post-extubation oxygenation strategies in acute respiratory failure: a systematic review and network meta-analysis

**DOI:** 10.1186/s13054-021-03550-4

**Published:** 2021-04-09

**Authors:** Hideto Yasuda, Hiromu Okano, Takuya Mayumi, Chihiro Narita, Yu Onodera, Masaki Nakane, Nobuaki Shime

**Affiliations:** 1 Department of Emergency and Critical Care Medicine, Jichi Medical University Saitama Medical Center, 1-847, Amanuma-cho, Oomiya-ku, Saitama-shi, Saitama, 330-8503 Japan; 2 Department of Clinical Research Education and Training Unit, Keio University Hospital Clinical and Translational Research Center (CTR), 35, Shinanomachi, Shinjuku-ku, Tokyo, 160-8582 Japan; 3 Department of Critical and Emergency Medicine, National Hospital Organization Yokohama Medical Center, 2-60-3, Harajyuku, Totsuka-ku, Yokohama-shi, Kanagawa, 245-8575 Japan; 4 Department of Cardiovascular Medicine, Graduate School of Medical Science, Kanazawa University, 1-13, Takaramachi, Kanazawa-shi, Ishikawa, 920-0934 Japan; 5 Department of Emergency Medicine, Shizuoka General Hospital, 1-27-4, Kitaandou, Aoi-ku, Shizuoka-shi, Shizuoka, 420-8527 Japan; 6 Department of Anesthesiology, Yamagata University Faculty of Medicine, 2-2-2, Iidanishi, Yamagata-shi, Yamagata, 990-2331 Japan; 7 Department of Emergency and Critical Care Medicine, Yamagata University Hospital, 2-2-2, Iidanishi, Yamagata-shi, Yamagata, 990-2331 Japan; 8 Department of Emergency and Critical Care Medicine, Postgraduate School of Medical Science, Hiroshima University Hospital, 3-2-1, Kasumi, Minami-ku, Hiroshima-shi, Hiroshima, 734-8551 Japan

**Keywords:** Post-extubation, Conventional oxygen therapy, Noninvasive ventilation, High-flow nasal cannula, Systematic review, Meta-analysis, Network meta-analysis

## Abstract

**Background:**

High-flow nasal cannula oxygenation (HFNC) and noninvasive positive-pressure ventilation (NPPV) possibly decrease tracheal reintubation rates better than conventional oxygen therapy (COT); however, few large-scale studies have compared HFNC and NPPV. We conducted a network meta-analysis (NMA) to compare the effectiveness of three post-extubation respiratory support devices (HFNC, NPPV, and COT) in reducing the mortality and reintubation risk.

**Methods:**

The Cochrane Central Register of Controlled Trials, MEDLINE, EMBASE, and Ichushi databases were searched. COT, NPPV, and HFNC use were assessed in patients who were aged ≥ 16 years, underwent invasive mechanical ventilation for > 12 h for acute respiratory failure, and were scheduled for extubation after spontaneous breathing trials. The GRADE Working Group Approach was performed using a frequentist-based approach with multivariate random-effect meta-analysis. Short-term mortality and reintubation and post-extubation respiratory failure rates were compared.

**Results:**

After evaluating 4631 records, 15 studies and 2600 patients were included. The main cause of acute hypoxic respiratory failure was pneumonia. Although NPPV/HFNC use did not significantly lower the mortality risk (relative risk [95% confidence interval] 0.75 [0.53–1.06] and 0.92 [0.67–1.27]; low and moderate certainty, respectively), HFNC use significantly lowered the reintubation risk (0.54 [0.32–0.89]; high certainty) compared to COT use. The associations of mortality with NPPV and HFNC use with respect to either outcome did not differ significantly (short-term mortality and reintubation, relative risk [95% confidence interval] 0.81 [0.61–1.08] and 1.02 [0.53–1.97]; moderate and very low certainty, respectively).

**Conclusion:**

NPPV or HFNC use may not reduce the risk of short-term mortality; however, they may reduce the risk of endotracheal reintubation.

***Trial registration number and date of registration*:**

PROSPERO (registration number: CRD42020139112, 01/21/2020).

**Supplementary Information:**

The online version contains supplementary material available at 10.1186/s13054-021-03550-4.

## Background

Invasive mechanical ventilation (IMV) is a life-saving procedure for patients with acute respiratory failure (ARF) [1]. Approximately 10–20% of the patients who are extubated after a successful spontaneous breathing trial (SBT) require reintubation within 48–72 h [2], which may be associated with prolonged mechanical ventilation, extended intensive care unit (ICU) and hospital stay, and increased mortality [2, 3].

Various oxygenation therapies have been proposed to prevent reintubation in ARF due to several causes, including hypoxia, ventilatory insufficiency, and increased respiratory workload. Conventional oxygen therapy (COT) and noninvasive positive-pressure ventilation (NPPV) have been recommended as post-extubation respiratory support devices [4–7]; recently, high-flow nasal cannula oxygenation (HFNC) has also been used as a prophylactic post-extubation respiratory support device to avoid reintubation [8, 9].

NPPV has been reported to be effective in preventing reintubation after planned extubation in high-risk patients [6, 7, 10, 11]. However, NPPV may increase the risk of complications, including aspiration pneumonia, interface intolerance, and patient discomfort [12, 13]. HFNC can minimize the complications of NPPV by delivering high concentrations of humidified oxygen via a nasal cannula. However, contradictory results have been reported despite the large number of clinical trials [14, 15].

Some systematic reviews and meta-analyses which compared two of the three respiratory support devices (COT, NPPV, and HFNC) [16–20] have shown that in terms of reducing the rate of tracheal reintubation, HFNC was better than COT but equivalent to NPPV. Moreover, there were no significant differences between the therapies in terms of mortality rates. Although several studies have compared HFNC and NPPV with COT, few large-scale studies have compared HFNC with NPPV. Therefore, small sample sizes may have affected the results of systematic reviews.

Therefore, we performed a systematic review and network meta-analysis (NMA) to compare the effectiveness of three respiratory support devices in reducing mortality and reintubation rates by including studies that compared two of the three respiratory support devices (COT, NPPV, and HFNC) in patients who were intubated for ARF after scheduled extubation.

## Methods

### Protocol and registration

This systematic review was designed in accordance with the Preferred Reporting Items for Systematic review and Meta-Analyses (PRISMA) extension statements for reporting systematic reviews that incorporate NMA (Additional file 1: Table S1) [21]. The review protocol was registered with PROSPERO (CRD42020139112).

### Studies, participants, interventions/comparators, and outcomes

We included all reports of randomized controlled trials (RCTs) in English and Japanese regardless of publication status (e.g., published, unpublished, and academic abstracts). Randomized crossover, cluster randomized, and quasi-experiment trials were excluded. This meta-analysis included reviews of adult patients (age ≥ 16 years) who underwent IMV for more than 12 h due to ARF and were scheduled for extubation after a SBT. The definitions of acute hypoxic respiratory failure and SBT were individualized for each study. This meta-analysis excluded studies that included patients who underwent tracheostomies, experienced accidental extubation or self-extubation, those who experienced hypercapnia during SBT, and those who had do-not-resuscitate (DNR) orders. Studies in which more than half of the study population had acute chronic obstructive pulmonary disease (COPD) exacerbation, those that included patients with a postoperative status or who were being treated for trauma, and those that included patients with congestive heart failure were also excluded. We included RCTs that compared two of the three available respiratory support devices: (1) COT: low-flow nasal cannula, face mask, and venturi mask (no flow rate restriction); (2) NPPV: the type of mask, mode, duration of ventilation, and weaning methods were not limited; and (3) HFNC: no limitations on the flow rate or F_I_O_2_. The outcome measures evaluated were as follows: the primary outcome was the short-term mortality rate ([1] at the end of the follow-up period for each trial within 30 days, [2] at ICU discharge, and [3] at hospital discharge). Secondary outcomes included the reintubation rate within 72 h (reintubation included the need for intubation and NPPV) and post-extubation respiratory failure rate (the definition was individualized for each study).

### Data sources and search details

We searched the Cochrane Central Register of Controlled Trials (CENTRAL), MEDLINE via PubMed, EMBASE, and Ichushi, a database of Japanese papers for eligible trials. We searched for ongoing trials in the World Health Organization International Clinical Trials Platform Search Portal. In cases of missing data, we attempted to contact the authors of each study. Searches were performed in December 2020. Details regarding search strategy and when the searches were performed are shown in Additional file 1: Table S2.

### Study selection, data collection process, and data items

Two of the three physicians (YO, CN, and HY) screened the title, abstract, and full text during the first and second screenings for relevant studies and independently extracted data from eligible studies into standardized data forms. For abstract-only studies that could not be evaluated according to the eligibility criteria, we contacted the authors. Disagreements, if any, between two reviewers were resolved via discussion among themselves or with a third reviewer as necessary. Data extraction from identified studies during the second screening was also performed by two of the three physicians (YO, CN, and HY) using two tools: (1) the Cochrane Data Collection Form (RCTs only) [22] and (2) Review Manager (RevMan) software V.5.3.5 [23]. Disagreements, if any, were resolved in the same manner as for the screening process.

### Risk of bias within individual studies

The risk of bias for primary outcomes was independently assessed by two of the three physicians (YO, CN, and HY) using the Cochrane Risk of Bias tool 1.0 [24, 25]. Each bias was graded as “low risk,” “unclear risk,” or “high-risk.” Discrepancies between reviewers were resolved by mutual discussion.

### Statistical analyses

#### Direct comparison meta-analysis

A pairwise meta-analysis was performed by using RevMan 5.3 (RevMan 2014). Forest plots were used for the meta-analysis, and effect sizes are expressed as relative risk (RR) and weighted mean differences, both with 95% confidence intervals (CI), for categorical and continuous data, respectively. Outcome measures were pooled using a random-effect model to include study-specific effects in measures. A two-sided *p* value < 0.05 was considered significant.

Study heterogeneity between trials for each outcome was assessed by visual inspection of forest plots and with an *I*^2^ statistic for quantifying inconsistency [26] (RevMan; *I*^2^: 0–40%, 30–60%, 50–90%, and 75–100% as minimal, moderate, substantial, and considerable heterogeneity, respectively). When heterogeneity was identified (*I*^2^ > 50%), we investigated the reason and quantified it using the Chi-square test (*p* value).

We planned to use a funnel plot, Begg’s adjusted rank correlation test, and Egger’s regression asymmetry test for the possibility of publication bias, if ≥ 10 studies were available (RevMan) [27]. However, as < 10 studies were included for each outcome, we did not test for funnel plot asymmetry.

#### Network comparison meta-analysis

##### Data synthesis

A network plot was constructed to determine the number of studies and patients included in this meta-analysis. An NMA, using the netmeta 0.9–5 R-package (version 3.5.1), was performed using a frequentist-based approach with multivariate random-effect meta-analysis, and effect size was expressed as the RR (95% CI). Covariance between two estimates from the same study shows variance of data in the shared arm, as calculated in a multivariable meta-analysis performed using the GRADE Working Group Approach for an NMA.

##### Transitivity

The transitivity assumption underlying the NMA was evaluated by comparing the distribution of clinical and methodological variables that could act as effect modifiers across treatment comparisons.

##### Ranking

Ranking plots (rankograms) were constructed using the probability that a given treatment had the highest event rate for each outcome. The surface under the cumulative ranking curve (SUCRA), which is a simple transformation of the mean rank, was used to set the hierarchy of the treatments [28] and was created using standard software (Stata 15.0, Stata, TX, USA).

##### Risk of bias across studies

Assessment of the risk of bias across studies followed considerations on pairwise meta-analysis. Conditions associated with “suspected” and “undetected” bias across studies were determined by the presence of publication bias as shown by direct comparison.

##### Indirectness

The indirectness of each study included in the network was evaluated according to its relevance to the research question, which consisted of the study population, interventions, outcomes, and study setting, and was classified as low, moderate, or high. Study-level judgments could be combined with the percentage contribution matrix.

##### Imprecision

The approach to imprecision comprised a comparison of the range of treatment effects included in the 95% CI with the range of equivalence. We assessed the heterogeneity of treatment effects for a clinically important risk ratio (< 0.8 or > 1.25) in CI.

##### Heterogeneity

To assess the amount of heterogeneity, we compared the posterior distribution of the estimated heterogeneity variance with its predictive distribution [29]. The concordance between assessments based on CI and prediction intervals, which do and do not capture heterogeneity, respectively, was used to assess the importance of heterogeneity. We assessed the heterogeneity of treatment effects for a clinically important risk ratio of < 0.8 or > 1.25 in prediction intervals.

##### Assessment of inconsistency

The inconsistency of the network model was estimated from inconsistency factors and their uncertainty, and consistency was statistically evaluated using the design-by-treatment interaction test [30]. For comparisons informed only by direct evidence, there was no disagreement between evidence sources, and thus, there was “no concern” for incoherence. If only indirect evidence was included, there was always “some concern.” “Major concern” was considered when the *p* value of the design-by-treatment interaction test was < 0.01.

## Results

### Study selection

**Fig. 1:**
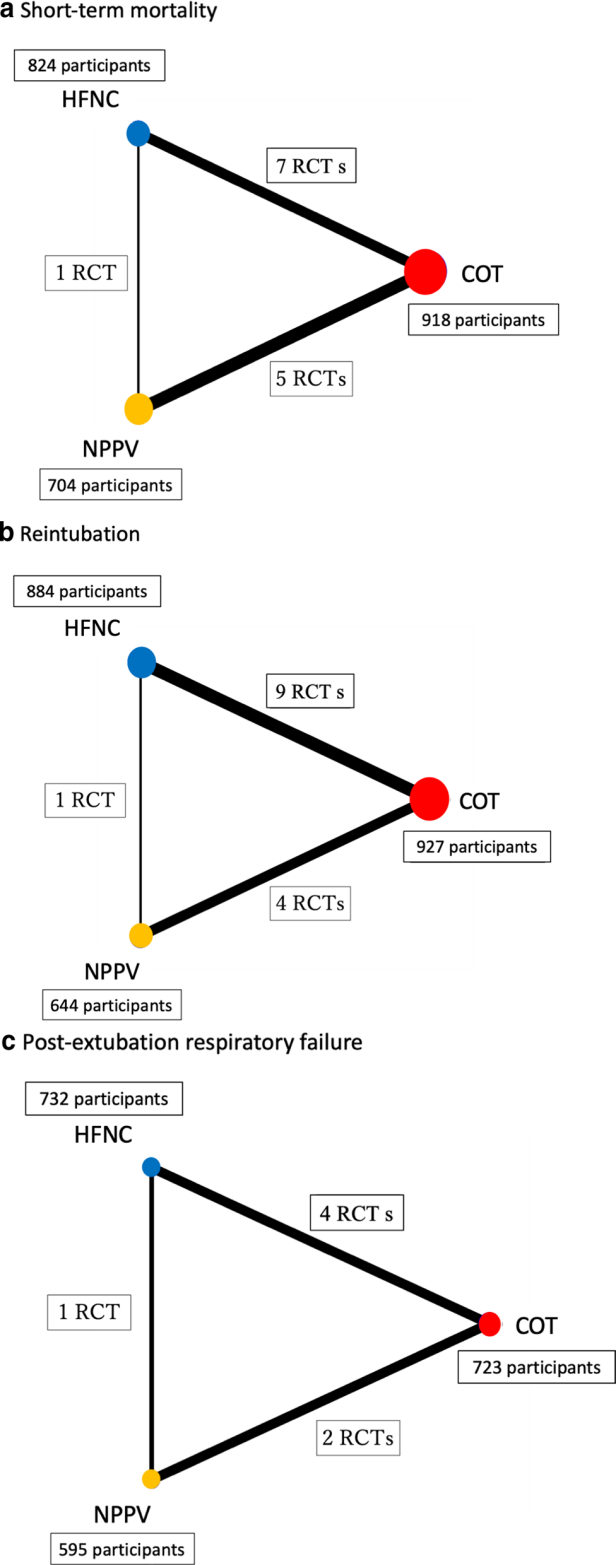
Network plots correlating noninvasive oxygenation strategies with short-term mortality, reintubation, post-extubation respiratory failure. **a** Short-term mortality. **b**. Reintubation, **c** post-extubation respiratory failure

A comprehensive search yielded a total of 4,631 records (Additional file 1: Fig. S1), from which 15 studies were included in this NMA [6, 14, 15, 31–42]. Of the 15 studies, one [35] was an abstract that was presented at a conference and not published elsewhere. None of the studies was a three-group study that directly compared NPPV with HFNC and COT; studies 5, 9, and 1 compared NPPV with COT, HFNC with COT, and HFNC with NPPV, respectively (network structures per outcome in Fig. 1).

### Study characteristics

The protocols and characteristics of each study included in this meta-analysis are summarized in Table 1. A total of 2,600 patients were included in the quantitative analysis. The main cause of acute hypoxic respiratory failure was pneumonia, followed by postoperative respiratory failure. Of the 15 studies, two mainly included patients with exacerbation of chronic respiratory disorders.

**Table 1:** Study populations, protocols, and study characteristics

References	Publication status	Sample size n	Protocols				Baseline characteristics		
			Intervention setting	Control setting	Outcomes	Age, years	PaO_2_:FiO_2_ ratio	Main reason for initiation of mechanical ventilation	Duration of intubation, days
Ferrer et al. [6]	Published	162	NPPV	COT	1. Mortality (in-hospital) 2. Reintubation 3. Respiratory failure	NPPV: 72 (10) COT: 70 (11)	NPPV: 278 (95) COT: 276 (94)	Exacerbation of chronic respiratory disorders [30.2%]	NPPV: 6 (4) COT: 7 (5)
Su et al. [33]	Published	406	NPPV	COT	1. Mortality (in-ICU) 2. Reintubation 3. Respiratory failure	NPPV: 65 (1) COT: 63 (1)	NA	Postoperative respiratory failure [24.4%]	NA
Mohamed and Abdalla [32]	Published	120	NPPV	COT	1. Mortality (in-ICU)	NPPV: 64 (7) COT: 69 (7)	NA	COPD [29.2%]	NPPV: 6.2 (1.6) COT: 7.1 (1.8)
Ornico et al. [31]	Published	38	NPPV	COT	1. Mortality (in-hospital) 2. Reintubation	NPPV: 51 (18) COT: 49 (22)	NA	Pneumonia [84.2%]	NPPV: 9.9 (8.1) COT: 9.5 (6.1)
Maggiore et al. [38]	Published	105	HFNC	COT	1. Mortality (in-ICU) 2. Reintubation	HFNC: 65 (18) COT 64 (17)	HFNC: 239 (42) COT: 242 (51)	Pneumonia [45.7%]	HFNC: 4.6 (4.1) COT: 5.2 (3.7)
Hernández [15]	Published	527	HFNC	COT	1. Mortality (in-hospital) 2. Reintubation 3. Respiratory failure	HFNC: 51 (13) COT: 52 (12)	HFNC: 227 (25) COT: 237 (34)	Urgent surgery [24.9%]	HFNC: 1 [1–3]^a^ COT: 2 [1–4]^a^
Hernandez et al. [36]	Published	604	NPPV	HFNC	1. Mortality (in-hospital) 2. Reintubation 3. Respiratory failure	NPPV: 64 (16) HFNC: 65 (15)	NPPV: 194 (37) HFNC: 191 (34)	Urgent surgery [31.5%]	NPPV: 4 [2–8]^a^ HFNC: 4 [2–9]^a^
Arman et al. [35]	Unpublished	15	HFNC	COT	1. Mortality (30 days) 2. Reintubation	NA	NA	AHRF	NA
Fernandez et al. [14]	Published	155	HFNC	COT	1. Mortality (in-ICU, In-hospital) 2. Reintubation 3. Respiratory failure	HFNC: 67 (12) COT: 70 (13)	NA	AHRF	HFNC: 8.2 (5.9) COT: 7.4 (3.6)
Song et al. [37]	Published	60	HFNC	COT	1. Reintubation	HFNC: 66 (14) COT 71 (13)	NA	Pneumonia [41.7%]	HFNC: 5.5 (3.4) COT: 5.4 (2.8)
Thanthitaweewat et al. [34]	Published	58	NPPV	COT	1. Mortality (28 days) 2. Reintubation	NPPV: 63 (22) COT: 63 (19)	NPPV: 330 (104) COT: 359 (179)	Pneumonia [58.6%]	NPPV: 4 [5]^a^ COT: 7 [7]^a^
Cho et al. [39]	Published	60	HFNC	COT	1. Mortality (in-ICU, In-hospital) 2. Reintubation	HFNC: 79 (8) COT 77 (7)	HFNC: 272 (99) COT 297 (119)	Pneumonia [66.7%]	HFNC: 7.1 (4.7) COT 5.7 (5.2)
Hu et al. [40]	Published	56	HFNC	COT	1. Mortality (in-hospital) 2. Reintubation 3. Respiratory failure	HFNC: 73 (13) COT 75 (11)	HFNC: 320 (90) COT 279 (91)	Pneumonia [39.3%]	HFNC: 9 [6]^a^ COT: 7 [4]^a^
Matsuda et al. [41]	Published	69	HFNC	COT	1. Reintubation	HFNC: 72 (18) COT 71 (16)	HFNC: 227 (43) COT 216 (37)	Pneumonia [53.6%]	HFNC: 5 (2) COT 6 (3)
Theerawit et al. [42]	Published	140	HFNC	COT	1. Mortality (in-hospital) 2. Reintubation 3. Respiratory failure	HFNC: 68 (19) COT 71 (16)	HFNC: 298 (96) COT 289 (114)	Pneumonia [59.3%]	HFNC: 6.9 (4.9) COT 6.2 (4.0)

*AHRF* acute hypoxic respiratory failure, *COPD* chronic obstructive pulmonary disease, *COT* conventional oxygen therapy, *ICU* intensive care unit, *HFNC* high-flow nasal cannula, *NPPV* noninvasive positive-pressure ventilation

Continuous data are shown as mean and standard deviation, except for data labeled with “a”

^a^Data reported as median and IQR (interquartile range)

### Risk of bias within studies

The risk of bias within included studies is shown in Additional file 1: Fig. S2. Although not all studies blinded participants and clinicians to the intervention, almost all other domains of the risk of bias were low (Additional file 1: Fig. S2). All studies were judged as having a low risk of bias for outcomes (risk of bias across studies).

### Network meta-analysis

The results of pairwise comparisons are shown in Additional file 1: Figs. S3, S4, and S5 (short-term mortality, reintubation, and post-extubation respiratory failure, respectively). The funnel plot of each outcome was not described because the number of studies included for each comparison was < 10.

#### Short-term mortality

**Fig. 2:**
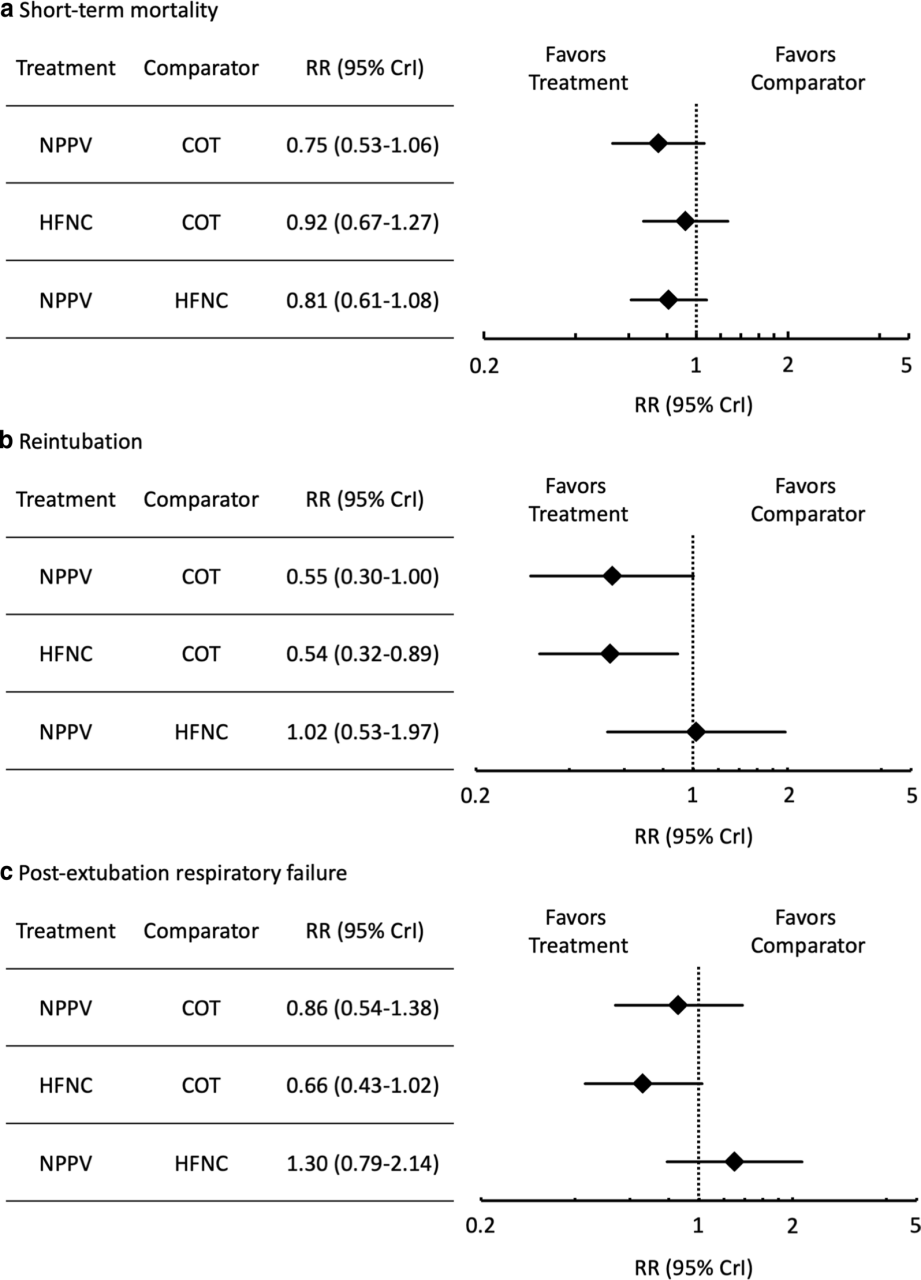
Network meta-analysis forest plots on noninvasive oxygenation strategies and short-term mortality, reintubation, post-extubation respiratory failure. **a**. Short-term mortality. **b**. Reintubation. **c** Post-extubation respiratory failure

Thirteen studies were included in the analysis of short-term mortality. Compared with COT, NPPV (RR 0.75 [95% CI 0.53–1.06]: low certainty) and HFNC (RR 0.92 [95% CI 0.67–1.27]: moderate certainty) were not associated with a lower risk of mortality (Fig. 2a). There was no significant difference in association with mortality between NPPV and HFNC (RR 0.81 [95% CI 0.61–1.08]: moderate certainty). Anticipated absolute effects and 95% CIs between two comparisons decreased by 26 per 1000 (95% CI − 49 to + 6) for NPPV vs. COT, 7 per 1000 (95% CI − 28 to + 23) for HFNC vs. COT and 39 per 1000 (95% CI − 79 to + 16) for NPPV vs. HFNC (Table 2).

**Table 2:**
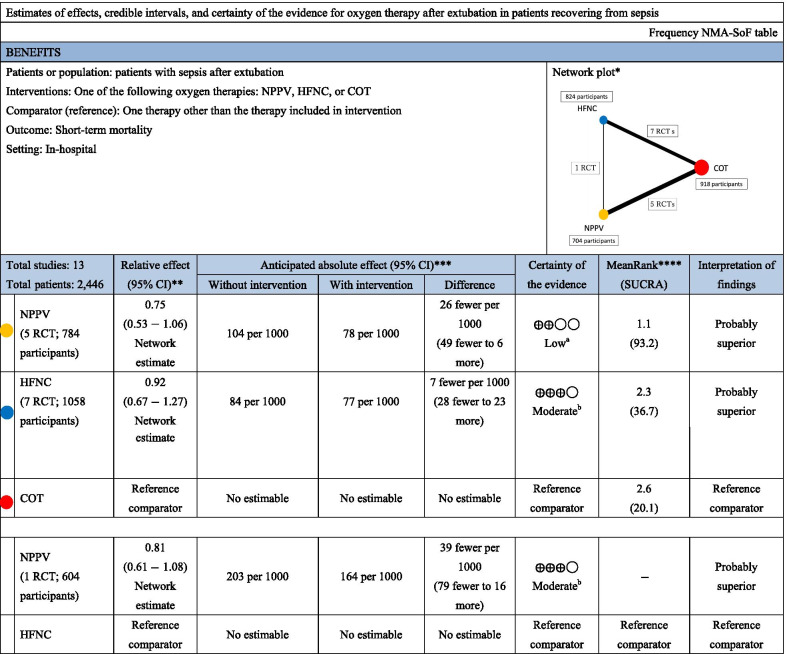
Summary of findings for short-term mortality from the network meta-analysis

*NMA* network meta-analysis, *NPPV* noninvasive positive-pressure ventilation, *HFNC* high-flow nasal cannula, *COT* conventional oxygen therapy, *SOF* summary of findings, *SUCRA* surface under the cumulative ranking

NMA-SoF table definitions

*Lines represent direct comparisons

**Estimates are reported as risk ratio. CI: confidence interval

***Anticipated absolute effect. Anticipated absolute effect compares two risks by calculating the difference between the risks in the intervention and control groups

****Rank for efficacy outcomes is presented. Rank statistics are defined as the probabilities that one treatment out of *n* treatments in a network meta-analysis is the best, the second best, the third best, and so on, until the least effective treatment

GRADE Working Group grades of evidence (or certainty in the evidence)

High quality: We are very confident that the true effect lies close to that of the estimate of the effect

Moderate quality: We are moderately confident in the effect estimate: The true effect is likely close to the estimate of the effect, but there is a possibility that it is substantially different

Low quality: Our confidence in the effect estimate is limited: The true effect may be substantially different from the estimate of the effect

Very low quality: We have very little confidence in the effect estimate: The true effect is likely to be substantially different from the estimate effect

Explanatory Footnotes

^a^Confidence interval extends into clinically important effects in both directions

^b^Confidence interval extends into clinically important effects

Confidence in the RR of each comparison and short-term mortality, assessed by the GRADE system, is shown in Table 3. Incoherence between direct and indirect RRs was not observed for any of the three comparisons, according to the *p* values of inconsistency. The heterogeneity for all three comparisons resulted in a “no concern” rating due to the 95% prediction interval of the risk ratio.

**Table 3:** Confidence in the relative risk of each comparison and outcome assessed by the GRADE system for short-term mortality, reintubation, and post-extubation respiratory failure

	Risk of bias across studies	Imprecision	Heterogeneity	Indirectness	Publication bias	Incoherence	Confidence in relative risk of the event
*Short-term mortality*							
NIV vs. COT	Undetected	Very serious^a^ (95% CI 0.53–1.06)	No concern (95% PI 0.51–1.11)	Low	Not suggested	No concern (p = 0.33)	⨁⨁◯◯ Low
HFNC vs. COT	Undetected	Serious^b^ (95% CI 0.67–1.27)	No concern (95% PI 0.64–1.33)	Low	Not suggested	No concern (p = 0.33)	⨁⨁⨁◯ Moderate
HFNC vs. NIV	Undetected	Serious^b^ (95% CI 0.61–1.08)	No concern (95% PI 0.58–1.13)	Low	Not suggested	No concern (p = 0.33)	⨁⨁⨁◯ Moderate
*Reintubation*							
NIV vs. COT	Undetected	Serious^b^ (95% CI 0.30–1.00)	Some concern^c^ (95% PI 0.16–1.84)	Low	Not suggested	No concern (p = 0.58)	⨁⨁⨁◯ Moderate
HFNC vs. COT	Undetected	Not serious (95% CI 0.32–0.89)	Major concern^d^ (95% PI 0.17–1.70)	Low	Not suggested	No concern (p = 0.58)	⨁⨁⨁⨁ High
HFNC vs. NIV	Undetected	Very serious^a^ (95% CI 0.53–1.97)	No concern (95% PI 0.29–3.55)	Low	Not suggested	No concern (p = 0.58)	⨁⨁◯◯ Low
*Post-extubation respiratory failure*							
NIV vs. COT	Undetected	Very serious^a^ (95% CI 0.54–1.38)	No concern (95% PI 0.29–2.58)	Low	Not suggested	No concern (p = 0.56)	⨁⨁◯◯ Low
HFNC vs. COT	Undetected	Serious^b^ (95% CI 0.43–1.02)	Some concern^c^ (95% PI 0.23–1.92)	Low	Not suggested	No concern (p = 0.56)	⨁⨁⨁◯ Moderate
HFNC vs. NIV	Undetected	Very serious^a^ (95% CI 0.79–2.14)	No concern (95% PI 0.42–3.98)	Low	Not suggested	No concern (p = 0.56)	⨁⨁◯◯ Low

*CI* confidence interval, *COT* conventional oxygen therapy, *HFNC* high-flow nasal therapy, *NIV* noninvasive ventilation, *PI* prediction interval

^a^Confidence interval extends into clinically important effects in both directions

^b^Confidence interval extends into clinically important effects

^c^Prediction interval extends into clinically important or unimportant effects

^d^Prediction interval extends into clinically important effects in both directions

**Fig. 3:**
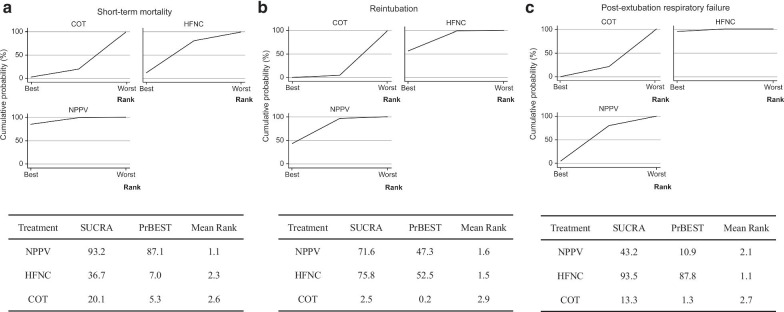
Surface under cumulative ranking of noninvasive oxygen strategies for short-term mortality, reintubation, post-extubated respiratory failure. **a** Short-term mortality. **b** Reintubation. **c**. Post-extubation respiratory failure

Figure 3a shows the ranking analysis results, which revealed that the hierarchy for efficacy in reducing short-term mortality was NPPV (SUCRA 93.2) > HFNC (SUCRA 36.7) > COT (SUCRA 20.1). Table 2 summarizes the findings of the NMA for short-term mortality. Moreover, Additional file 1: Table S3 summarizes the estimate and certainty of the evidence of direct, indirect, and network comparisons.

#### Endotracheal reintubation

Fourteen studies were included in the analysis of endotracheal reintubation. Compared with COT, HFNC (RR 0.54 [95% CI 0.32–0.89]: high certainty) was significantly associated with a lower risk of reintubation (Fig. 2b), although there was no significant difference in association with reintubation between NPPV and HFNC (RR 1.02 [95% CI 0.53–1.97]: low certainty) and between NPPV and COT (RR 0.55 [95% CI 0.30–1.00]: moderate certainty). Anticipated absolute effects and 95% CIs between each of the two comparisons decreased by 62 per 1000 (95% CI − 96 to 0) for NPPV vs. COT and 60 per 1000 (95% CI − 88 to − 14) for HFNC vs. COT, but increased by 5 per 1000 (95% CI − 107 to + 221) for NPPV vs. HFNC (Table 4).

**Table 4:**
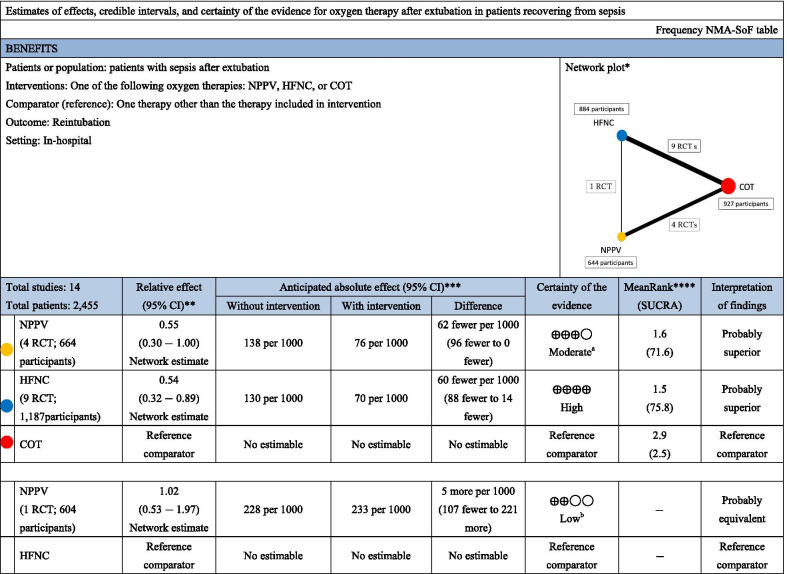
Summary of findings for reintubation from the network meta-analysis

*NMA* network meta-analysis, *NPPV* noninvasive positive-pressure ventilation, *HFNC* high-flow nasal cannula, *COT* conventional oxygen therapy, *SOF* summary of findings, *SUCRA* surface under the cumulative ranking

NMA-SoF table definitions

^*^Lines represent direct comparisons

^**^Estimates are reported as risk ratio. CI: confidence interval

^***^Anticipated absolute effect. Anticipated absolute effect compares two risks by calculating the difference between the risks in the intervention and control groups

^****^Rank for efficacy outcomes is presented. Rank statistics are defined as the probabilities that one treatment out of *n* treatments in a network meta-analysis is the best, the second best, the third best, and so on, until the least effective treatment

GRADE Working Group grades of evidence (or certainty in the evidence)

High quality: We are very confident that the true effect lies close to that of the estimate of the effect

Moderate quality: We are moderately confident in the effect estimate: The true effect is likely close to the estimate of the effect, but there is a possibility that it is substantially different

Low quality: Our confidence in the effect estimate is limited: The true effect may be substantially different from the estimate of the effect

Very low quality: We have very little confidence in the effect estimate: The true effect is likely to be substantially different from the estimate effect

Explanatory Footnotes

^a^Confidence interval extends into clinically important effects

^b^Confidence interval extends into clinically important effects in both directions

Table 3 shows the confidence in the RR of each comparison and reintubation assessed by the GRADE system. Incoherence between direct and indirect RRs was not observed for all three comparisons and was decided by the *p* value of inconsistency. The heterogeneity of two comparisons (NPPV vs. COT and HFNC vs. COT) resulted in “some concern” and “major concern,” but that of one comparison (HFNC vs. NPPV) resulted in a “no concern” rating due to the 95% prediction interval of the risk ratio.

Figure 3b indicates the ranking analysis of the hierarchy for efficacy in reducing reintubation: HFNC (SUCRA 75.8) > NPPV (SUCRA 71.6) > COT (SUCRA 2.5). Table 4 summarizes findings of the NMA for reintubation; Additional file 1: Table S3 presents the estimate and certainty of the evidence of direct, indirect, and network comparisons.

#### Post-extubation respiratory failure

Seven studies were included in the analysis of post-extubation respiratory failure. Compared with COT, NPPV (RR 0.86 [95% CI 0.54–1.38]: low certainty) and HFNC (RR 0.66 [95% CI 0.43–1.02]: moderate certainty) were not associated with a lower risk of post-extubation respiratory failure (Fig. 2c). There was no significant difference in association with mortality between NPPV and HFNC (RR 1.30 [95% CI 0.79–2.14]: low certainty). Anticipated absolute effects and 95% CIs between each of the two comparisons decreased by 26 per 1000 (95% CI − 87 to + 71) for NPPV vs. COT and 57 per 1000 (95% CI − 95 to + 3) for HFNC vs. COT, but increased by 81 per 1000 (95% CI − 56 to + 307) for NPPV vs. HFNC (Table 5).

**Table 5:**
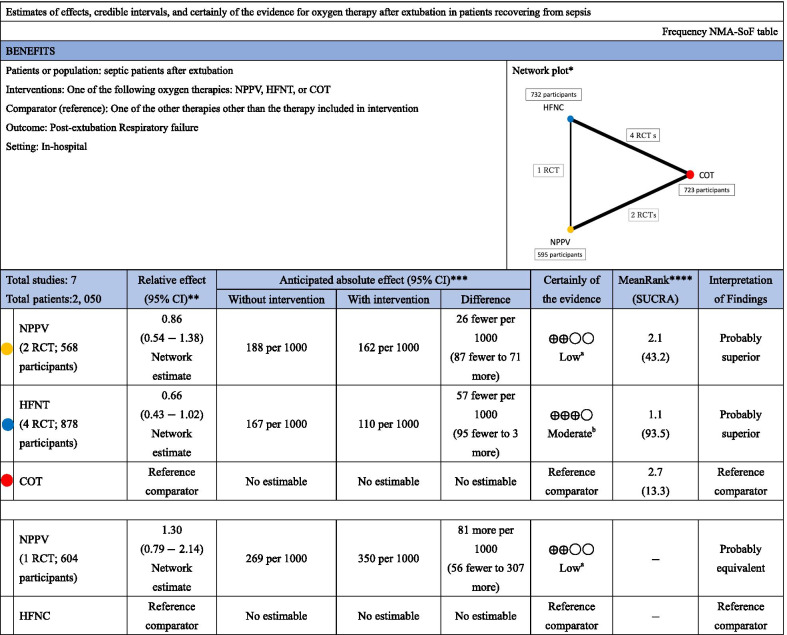
Summary table of findings in the network meta-analysis for post-extubation respiratory failure

*NMA* network meta-analysis, *NPPV* noninvasive positive-pressure ventilation, *HFNC* high-flow nasal cannula, *COT* conventional oxygen therapy, *SOF* summary of findings, *SUCRA* surface under the cumulative ranking

NMA-SoF table definitions

^*^Lines represent direct comparisons

^**^Estimates are reported as risk ratio. CI: confidence interval

^***^Anticipated absolute effect. Anticipated absolute effect compares two risks by calculating the difference between the risks of the intervention group with the risk of the control group

^****^Rank for efficacy outcome is presented. Rank statistics is defined as the probabilities that a treatment out of *n* treatments in a network meta-analysis is the best, the second, the third and so on until the least effective treatment

GRADE Working Group grades of evidence (or certainly in the evidence)

High quality: We are very confident that the true effect lies close to that of the estimate of the effect

Moderate quality: We are moderately confident in the effect estimate: The true effect is likely close to the estimate of the effect, but there is a possibility that it is substantially different

Low quality: Our confidence in the effect estimate is limited: The true effect may be substantially different from the estimate of the effect

Very low quality: We have very little confidence in the effect estimate: The true effect is likely to be substantially different from the estimate effect

Explanatory footnotes

^a^Confidence interval extends into clinically important effects in both directions

^b^Confidence interval extends into clinically important effects

Table 3 shows the confidence in the RR of each comparison and post-extubation respiratory failure assessed by the GRADE system. Incoherence between direct and indirect RRs was not observed for all three comparisons, as indicated by the *p* value of inconsistency. The heterogeneity of one comparison (HFNC vs. COT) and that of two comparisons (NPPV vs. COT and HFNC vs. NPPV) resulted in “some concern” and “no concern” ratings due to the 95% prediction interval of the risk ratio.

Figure 3c shows the results of the ranking analysis of the hierarchy for efficacy in reducing post-extubation respiratory failure: HFNC (SUCRA 93.5) > NPPV (SUCRA 43.2) > COT (SUCRA 13.3). Table 5 summarizes findings of the NMA for post-extubation respiratory failure, and Additional file 1: Table S3 summarizes the estimate and certainty of the evidence of direct, indirect, and network comparisons.

## Discussion

In our NMA, there were no between-group differences in short-term mortality (groups: NPPV, HFNC, and COT). NPPV/HFNC use did not significantly lower the mortality risk compared to COT use. The SUCRA value of short-term mortality for HFNC was better than those for NPPV and COT. However, as a secondary outcome, the use of HFNC significantly lowered the reintubation risk relative to COT use but not NPPV use. In addition, the SUCRA values of the reintubation rate and post-extubation respiratory failure for HFNC, NPPV, and COT use showed that HFNC use was superior to NPPV and COT use.

When HFNC was compared to COT, differences in outcomes between previous pairwise systematic reviews and this NMA-based study were observed. A systematic review by Ni and colleagues showed that HFNC is associated with a lower reintubation rate than COT, despite no reduction in mortality rate [16], which is identical to our study. Although a systematic review by Zhu et al. revealed that HFNC contributed to a reduction in post-extubation respiratory failure compared to that observed with COT, reductions in reintubation and mortality rates were not apparent [17]. In the study by Zhu et al. and our NMA, the effect of HFNC differed in terms of the reintubation rate; however, this difference is likely attributable to the eligibility of included patients. We excluded RCTs that included > 50% of postoperative patients, whereas the study by Zhu et al. included all RCTs with postoperative patients (three RCTs; *n* = 715) [43]. In postoperative abdominal surgery patients, diaphragmatic dysfunction and decreased lung vital capacity can cause atelectasis, resulting in hypoxemic respiratory failure [44]. Including patients with such different mechanisms of respiratory failure may increase patient heterogeneity and result in different outcomes compared to those observed with HFNC use and COT.

Herein, NPPV contributed to a reduction in the reintubation rate compared to that observed with COT, without reducing mortality, which is consistent with several previous pairwise systematic reviews comparing NPPV and COT use. Previous RCTs show that NPPV is more effective in reducing reintubation and mortality rates than COT in a high-risk group of patients with post-extubation respiratory failure, including COPD [7, 45, 46]. However, Kondo et al. showed that NPPV decreased reintubation and mortality rates more effectively than COT despite the complete exclusion of patients with COPD from the study [47]. In our study, we excluded studies in which patients with COPD constituted > 50% of the study population, as COPD is a risk factor for post-extubation respiratory failure [48]. Thus, the abovementioned exclusion potentially caused a difference between the effectiveness of NPPV and COT in the systematic reviews included in the NMA.

Zhou et al. recently reported a systematic review using NMA that compared NPPV, HFNC, and COT in post-extubation patients [49], but their inclusion criteria differ from ours. Zhou et al. included all studies with patients with COPD, whereas we excluded studies with > 50% COPD patients. Moreover, Zhou et al. showed that NPPV was associated with reductions in mortality and post-extubation respiratory failure rates compared to COT. COPD is a risk factor for reintubation after extubation and predisposes patients to hypercapnia during SBT [46]. Thus, NPPV is more effective than COT for patients with hypercapnia after extubation [50], which possibly led to differences in results between our study and that of Zhou et al. Furthermore, including trials with many patients with COPD potentially increased the patient heterogeneity. Therefore, we excluded trials where COPD patients accounted for > 50% of the study population. This study utilized a four-step approach for assessing the certainty of the NMA estimate developed by the GRADE Working Group [51], whereas the study by Zhou et al. did not conduct a similar assessment. A systematic approach using the GRADE system is necessary for evaluating the quality of the evidence to assess whether the evidence is convincing or of low quality, thereby guiding subsequent decision making.

### Implications

The results of our systematic review are useful for selecting an appropriate noninvasive oxygenation strategy for post-extubation patients because the use of NPPV or HFNC will prevent reintubation in a greater proportion of patients (66–69 patients per 1000) than the use of COT. Early weaning from IMV improves patient mortality, whereas reintubation significantly increases mortality risk [3]. Therefore, it is important to choose an appropriate strategy to prevent reintubation after extubation. Both NPPV and HFNC are associated with a lower reintubation rate than COT; therefore, physicians can choose a strategy according to the patient’s respiratory physiology status and preference.

### Limitations

Our systematic review using NMA has several limitations. First, we combined studies that included patients with different etiological conditions necessitating intubation, which may have increased the heterogeneity of the studies. Despite excluding RCTs with > 50% of patients with postoperative intubation and COPD, the inclusion of a fixed number of postoperative and COPD patients may have influenced the results. Second, we combined studies with different degrees of respiratory failure during the extubation of patients. The effect of NPPV and HFNC may differ depending on illness severity, and differences in severity may be an effect modifier. This NMA included other RCTs, with different characteristics, such as duration of intubation, risk factors for reintubation after extubation, and methods of SBT, which may also be effect modifiers. Third, because only one RCT directly compared NPPV and HFNC, there may not have been a significant difference due to insufficient sample size; there were no significant differences in mortality or post-extubation respiratory failure rates, but this may have been different if the sample size was larger. There was incoherence between direct and indirect estimation in the pairwise comparison of NPPV and HFNC, which led to a grading down of network estimation due to the lack of RCTs that directly compared NPPV and HFNC.

## Conclusion

In conclusion, noninvasive respiratory support strategies may not reduce the risk of short-term mortality compared with the use of COT; however, they may be associated with a lower reintubation rate.

## Supplementary Information

**Additional file 1: Table S1**: PRISMA NMA Checklist of Items to Include When Reporting A Systematic Review Involving a Network Meta-analysis. **Table S2**: Search strategy. **Table S3**: Estimate and certainty of the evidence of direct, indirect, and network comparison. (a) Short-term mortality. (b) Reintubation, (c) Post-extubation respiratory failure. **Fig. S1**: PRISMA Flow Diagram (search, inclusion, and exclusion). **Fig. S2**: Risk of bias summary for each comparison. (a) NPPV vs. COT. (b) HFNC vs. COT. (c) HFNC vs. NPPV. COT: conventional oxygen therapy, HFNC: high-flow nasal cannula oxygen; NPPV: noninvasive positive-pressure ventilation. **Fig. S3**: Forest plots for the pairwise comparison of short-term mortality. (a) NPPV vs. COT. (b) HFNC vs. COT. (c) HFNC vs. NPPV. COT: conventional oxygen therapy, HFNC: high-flow nasal cannula oxygen; NPPV: noninvasive positive-pressure ventilation. **Fig. S4**: Forest plots for the pairwise comparison of reintubation. (a) NPPV vs. COT. (b) HFNC vs. COT. (c) HFNC vs. NPPV. COT: conventional oxygen therapy, HFNC: high-flow nasal cannula oxygen; NPPV: noninvasive positive-pressure ventilation. **Fig. S5**: Forest plots for the pairwise comparison of post-extubated respiratory failure. (a) NPPV vs. COT. (b) HFNC vs. COT. (c) HFNC vs. NPPV. COT: conventional oxygen therapy, HFNC: high-flow nasal cannula oxygen; NPPV: noninvasive positive-pressure ventilation.

## Data Availability

The datasets generated during and/or analyzed during the current study are not publicly available due to post hoc analyses by co-authors but are available from the corresponding author on reasonable request.

## Abbreviations

ARF: Acute respiratory failure
CENTRAL: Cochrane Central Register of Controlled Trials
CI: Confidence interval
COPD: Chronic obstructive pulmonary disease
COT: Conventional oxygen therapy
DNR: Do-not-resuscitate
ICU: Intensive care unit
HFNC: High-flow nasal cannula oxygenation
IMV: Invasive mechanical ventilation
NPPV: Noninvasive positive-pressure ventilation
NMA: Network meta-analysis
PRISMA: Preferred Reporting Items for Systematic review and Meta-Analyses
RCT: Randomized controlled trial
RevMan: Review Manager
RR: Relative risk
SBT: Spontaneous breathing trial
SUCRA: Surface under the cumulative ranking curve
